# Changes of Immune Cell Fractions in Patients Treated with Immune Checkpoint Inhibitors

**DOI:** 10.3390/cancers14143440

**Published:** 2022-07-15

**Authors:** Hye Ryeon Kim, Jung Hun Kang, Sung Hyun Kim, Seung Tae Kim, Ilhwan Kim, Young Joo Min, Seong Hoon Shin, Sung Yong Oh, Gyeong-Won Lee, Ji Hyun Lee, Jun Ho Ji, Seok Jae Huh, Suee Lee

**Affiliations:** 1 Department of Internal Medicine, Dong-A University College of Medicine, Busan 49201, Korea; hyeryeon13@gmail.com (H.R.K.); kshmoon@dau.ac.kr (S.H.K.); drosy@dau.ac.kr (S.Y.O.); hidrleejh@dau.ac.kr (J.H.L.); doctorhsj@dau.ac.kr (S.J.H.); 2 Division of Hematology-Oncology, Department of Internal Medicine, Gyeongsang National University Hospital, Gyeongsang National University College of Medicine, Jinju 52727, Korea; newatp@naver.com (J.H.K.); brightree24@gmail.com (G.-W.L.); 3 Division of Hematology-Oncology, Department of Medicine, Samsung Medical Center, Sungkyunkwan University School of Medicine, Seoul 06351, Korea; seungtae1.kim@samsung.com; 4 Division of Hematology-Oncology, Department of Internal Medicine, Haeundae Paik Hospital, Inje University College of Medicine, Busan 48108, Korea; onelement@hanmail.net; 5 Division of Hematology-Oncology, Department of Internal Medicine, Ulsan University Hospital, University of Ulsan College of Medicine, Ulsan 44033, Korea; yjmin65@gmail.com; 6 Division of Medical Oncology, Department of Internal Medicine, Kosin University Gospel Hospital, Busan 49267, Korea; ssh1533@hanmail.net; 7 Division of Hematology and Oncology, Samsung Changwon Hospital, Sungkyunkwan University School of Medicine, Changwon 51353, Korea; junofanclub@hanmail.net

**Keywords:** immune checkpoint inhibitor, immune cell fraction, response rate, progression-free survival, overall survival

## Abstract

**Simple Summary:**

Immune checkpoint inhibitors (ICIs) are currently widely used in many types of cancer. However, some patients could not benefit from ICIs. In addition, a standardized biomarker for predicting the efficacy of ICIs is currently unavailable. Thus, we determined whether peripheral blood immune cells could be predicting markers. In contrast with previous studies, we focused on changes in immune cell fraction and the relationship between efficacy of ICIs including response rate and survival outcomes. Results revealed significant correlations between changes in NKp46-/CD56+ NK cell fraction and treatment outcomes with ICIs.

**Abstract:**

Background: With the development of immunology, immune checkpoint inhibitors (ICIs) have been widely used in various cancer treatments. Although some patients can benefit from ICIs, other patients have no response to ICIs or suffer from hyperprogression. There has been no biomarker for predicting the efficacy of ICIs. Thus, the objective of this study was to find biomarkers for predicting the efficacy of ICIs using peripheral blood. Methods: Adults patients planned to be treated with ICIs were enrolled in this study. Blood sampling was carried out before and after administration of ICIs. Changes of immune cell fraction were analyzed for each patient. Results: Among 182 patients enrolled, immune cell analysis was performed for 90 patients. The objective response rate was 14.4% (*n* = 13/90). The median progression-free survival (PFS) was 6.0 months (95% CI: 3.1–8.9 months), and the median overall survival (OS) was 13.9 months (95% CI: 5.6–22.2 months). Significant benefits in ORR and OS were shown for patients with increased NKp46-/CD56+ NK cells (*p* = 0.033 and *p* = 0.013, respectively). The PFS tended to be longer in these patients, although the difference was not statistically significant (*p* = 0.050). Conclusion: Changes of immune cell fraction before and after administration of ICIs could be a novel biomarker for predicting the efficacy of immunotherapy.

## 1. Introduction

Increasing knowledge of molecular and tumor biology has notably changed the paradigm of cancer treatment. Formerly, cancer was classified and treated according to organs of the origin or simplistic histomorphologic features. However, cancer treatment based on broad use of cytotoxic chemotherapies in unselected patients has reached its therapeutic plateau [1,2]. Thanks to molecular/cellular and genetic engineering studies, targeted therapy and monoclonal antibodies have been developed, significantly improving the survival and quality of life of cancer patients [3].

The development of immunology has led to the development of immune checkpoint inhibitors (ICIs). ICIs are humanized monoclonal antibodies that can activate T cells and relieve the immune system to recognize and assault cancer cells by targeting cytotoxic T-lymphocyte-associated protein 4 (CTLA4) (CD152), programmed cell death protein 1 (PD-1), or programmed cell death ligand-1 (PD-L1) [4]. These ICIs have improved the overall survival (OS) of patients with various cancer types and have become an important tool for cancer treatment [5,6,7]. However, only a subset of patients treated with ICIs experience durable clinical responses, whereas some patients suffer from hyper-progression, early death, and/or immune-related adverse events (AEs) [8]. Thus, there is a need to find predictive biomarkers that can further establish which patients are most likely to benefit from such therapies.

Candidates for predicting the efficacy of ICIs include PD-L1 expression assessed by immunohistochemistry (IHC), tumor mutation burden (TMB), mismatch repair deficiency (dMMR), and microsatellite instability-high (MSI-H). PD-L1 expression has been used as a biomarker for ICIs [9,10]. However, this alone has been insufficient to predict patient population most likely to respond to ICIs [10,11,12]. TMB is a measure of the number of mutations in cancer. It was first recognized as a potential biomarker for ICIs in melanoma [13,14]. Since then, many studies have reported a relationship between higher TMB and the efficacy of ICIs, suggesting that TMB could be a good predictive biomarker [13,14]. However, TMB is not significantly correlated with most cancer subtypes. It is not always correlated with responsiveness to ICIs [15]. dMMR and MSI-H have been suggested to be a novel predictor for anti-PD-1/PD-L1 immunotherapy efficacy by Zhao et al. [16]. However, only a fraction of patients typically possess dMMR/MSI-H features. Some sensitive patients still could not be distinguished [16]. TMB and dMMR/MSI-H status also have the issue of sampling, especially in a refractory/relapsed disease.

Previous studies have also used immune cells to predict the efficacy of ICIs and shown meaningful results. Peripheral blood analysis is a noninvasive method with good potential to predict treatment outcomes after immune therapies [17]. With an advantage in sampling, we conducted this pilot study to evaluate the possibility of change of immune cell fraction as a biomarker for predicting the efficacy of ICIs in cancer treatment.

## 2. Materials and Methods

### 2.1. Patients

This study was conducted at six sites in South Korea. Eligible patients were 18 years of age or older, with an Eastern Cooperative Oncology Group (ECOG) performance status of 0–2. Patients who were planned to be treated with ICIs (atezolizumab, nivolumab, pembrolizumab, or durvalumab) monotherapy or combination with chemotherapy for solid tumors or Hodgkin lymphoma were enrolled in this study. Patients who were previously exposed to PD-1, PD-L1, or CTLA4 inhibitors were excluded.

### 2.2. Immune Cell Analysis

**Figure A1:**
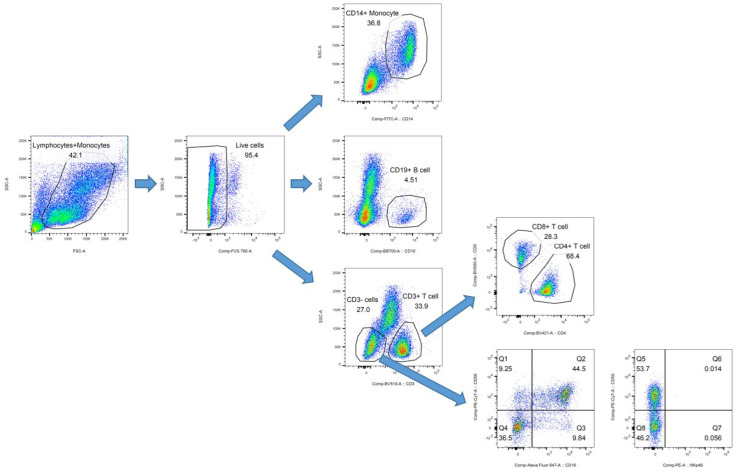
Immune cell analysis protocol (panel 1).

**Figure A2:**
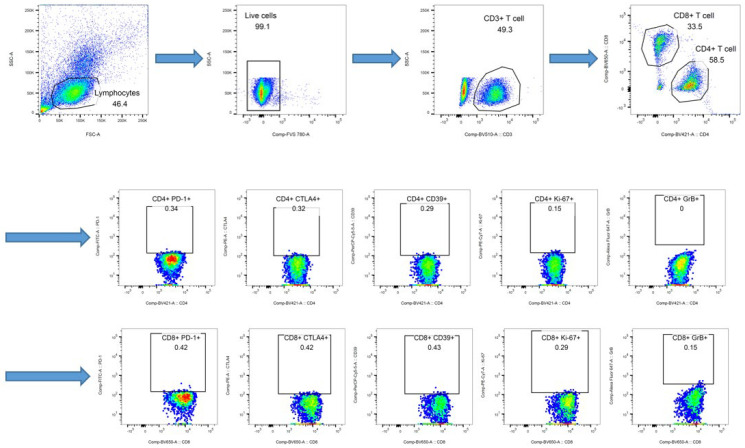
Immune cell analysis protocol (panel 2).

Peripheral blood sampling was carried out twice for each patient, before and after administration of ICIs. Pre-sampling was carried out just before the first cycle of treatment. Post-sampling was carried out when response evaluation was performed. Samples were sent to the lab for staining and analysis. Immune cells were divided into monocytes (CD14+), B cells (CD19+), T cells (CD3+, CD4+ or CD8+), and NK cells (CD3−) in panel 1 (Figure A1). In panel 2, CD4+ or CD8+ T cells were divided into exhausted (PD-1+, CTLA-4+, or CD39+), proliferation (Ki-67+), and effective (Granzyme B+) T cells (Figure A2). Immune cell analysis was performed by Sillajen Inc (Seoul, Korea) using a Muse^TM^ Cell Analyzer to analyze counts and viability of PBMC. The immune cell analysis protocol is described in the Appendix A. Antibodies used in the analysis are described in Table A1. Amount of surface staining reagents for panels 1 and 2 are described in Table A2 and Table A3, respectively.

### 2.3. Response and Survival Analysis

Tumor response assessments were performed based on Response Evaluation Criteria in Solid Tumors (RECIST) 1.1. The objective response rate (ORR) included complete response (CR) and partial response (PR). The clinical benefit rate (CBR) included CR, PR, and stable disease (SD). For patients who could not take follow-up imaging, the disease response assessment was performed according to the level of tumor marker and/or clinical features. Progression-free survival (PFS) was defined as the time from enrollment to objective tumor progression or death or was censored at the last radiographic assessment for patients without progression or death. OS was defined as the time from enrollment to death from any cause or was censored at the last follow-up date for patients who had follow-up loss due to any cause. Survival analyses were performed using the Kaplan-Meier method. Comparison was performed by the log-rank test and Fisher’s exact test. *p*-value of less than 0.05 was considered statistically significant. All statistical analyses were performed using IBM SPSS statistics version 27 (Armonk, NY, USA).

## 3. Results

### 3.1. Patients and Immune Cell Analysis

**Figure 1:**
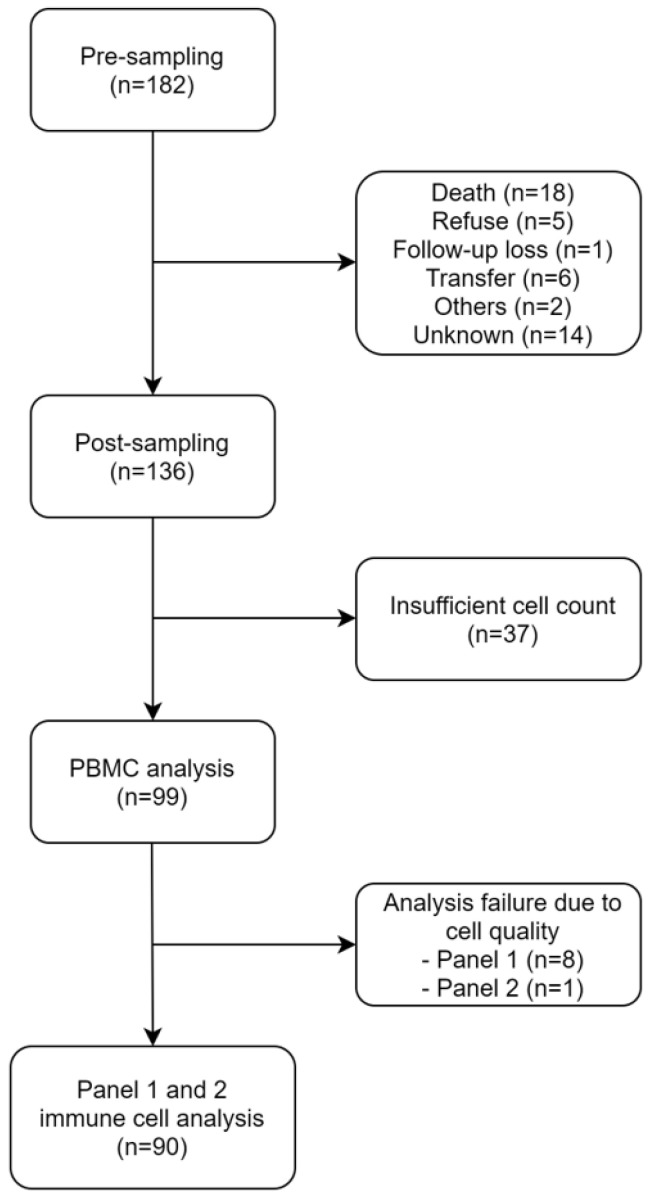
A flow chart of patient selection.

From December 2018 to April 2019, 178 patients were enrolled in this prospective study. Pre-sampling was carried out for all these patients. Post-sampling was carried out for 136 patients. The other 46 patients dropped out due to several causes: deaths *(n =* 18), refusal (*n* = 5), follow-up loss (*n* = 1), transfer to other hospitals (*n* = 6), other causes (*n* = 2), and unknown causes (*n* = 14). Cell count was insufficient to analyze in 37 patients. Thus, PBMC analysis was performed for 99 patients. However, analysis failed in nine patients owing to poor cell quality (eight in panel 1, one in panel 2). Finally, immune cell analysis (both panels 1 and 2) was performed for 90 patients (Figure 1).

The median age was 65 years old (range, 26 to 84 years). There were 55 (61.1%) males and 35 (38.9%) females. Here, 10 (11.1%), 78 (86.7%), and 2 (2.2%) patients had ECOG PS 0, 1, and 2, respectively. The most common type of cancer was lung cancer (50.0%, *n* = 45), followed by cholangiocarcinoma (7.8%, *n* = 7) and hepatocellular carcinoma (6.7%, *n* = 6). In this case, 13 (14.4%) patients were treatment-naïve and 14 (15.6%) patients were previously treated with more than three lines of therapy. During this study, the most common used ICI was nivolumab (44.4%, *n* = 40), followed by pembrolizumab (40.0%, *n* = 36) and atezolizumab (15.6%, *n* = 14). Two (2.2%) patients were treated with a combination therapy of pembrolizumab, pemetrexed, and cisplatin for lung cancer (Table 1).

Changes of each immune cell fraction are described in Table 2. Median values of change were −1.95% (range, −36.20 to 31.60) and 1.55% (range, −27.40 to 32.50) for CD4+ and CD8+ T cells, respectively. CD14+ monocytes and CD19+ B cells were generally decreased with median values of changes of −0.51% (range, −34.90% to 43.60%) and −0.27% (range, −6.90% to 10.38%), respectively. In NK cells, CD16+/CD56+ NK cells were generally increased with a median value of change of 4.05% (range, −37.10% to 48.58%), whereas CD16−/CD56− NK cells were generally decreased with a median value of change of −4.00% (range, −49.60% to 42.80%). PD-1+ CD4+ and PD-1+ CD8+ T cells were decreased with median values of changes of −5.49% (range, −30.60% to 24.77%) and −5.31% (range, −31.47% to 42.57%), respectively.

### 3.2. Response to Treatment and Survival Outcomes

**Figure 2:**
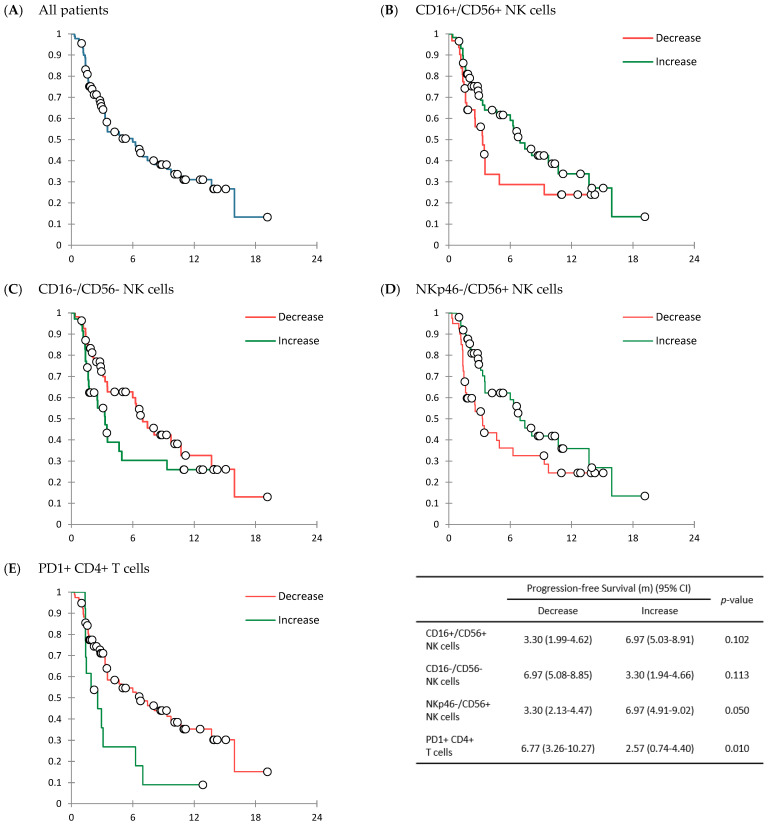
Kaplan Meier curves showing progression-free survival (PFS). PFS in all patients (*n* = 90) (**A**). PFS comparison according to the changes in immune cell fraction; CD16+/CD56+ NK cells (**B**), CD16−/CD56− NK cells (**C**), NKp46−/CD56+ NK cells (**D**), and PD-1+ CD4+ T cells (**E**).

**Figure 3:**
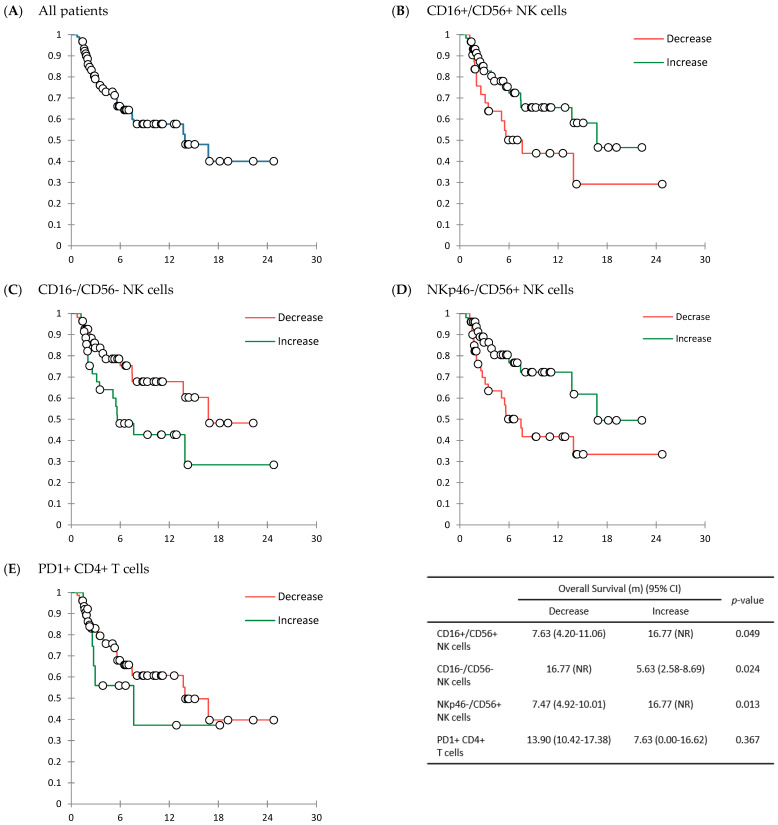
Kaplan Meier curves showing overall survival (OS). OS in all patients (*n* = 90) (**A**). OS comparison according to the changes in immune cell fraction; CD16+/CD56+ NK cells (**B**), CD16−/CD56− NK cells (**C**), NKp46−/CD56+ NK cells (**D**), and PD-1+ CD4+ T cells (**E**). Abbreviations: NR, no reached.

With a median follow-up duration of 4.63 months (IQR, 2.03 to 8.87 months), the median treatment cycle was 5 (range, 1 to 28), and the median treatment duration was 2.40 months (IQR, 1.31 to 5.53 months). Among 90 patients, no patient achieved CR. The ORR was 14.4% (*n* = 13) and the CBR was 50.0% (*n* = 45), including 14.4% for PR (*n* = 13) and 35.6% for SD (*n* = 32). In this case, 34 (37.8%) patients had progressive disease (PD). The response was not assessable for 11 (12.2%) patients. In 90 patients, the median PFS was 6.00 months (95% CI: 3.11 to 8.89 months), and the median OS was 13.90 months (95% CI: 5.62 to 22.19 months) (Table 3, Figure 2A and Figure 3A).

Response rate and survival results were compared by dividing patients into two groups: an increase in each immune cell fraction and a decrease in each immune cell fraction. In ORR, there was a statistically significant difference according to the change in NKp46-/CD56+ NK cells. The ORR was 5.0% (*n* = 2/40) in patients with a decrease in NKp46−/CD56+ NK cells and 22.0% (*n* = 11/50) (*p* = 0.033) in patients with an increase in NKp46-/CD56+ NK cells. Except for NKp46-/CD56+ NK cells, there was no significant difference in ORR or CBR by the change in immune cell fractions (Table S1).

In survival results, there were several notable differences in PFS (Figure 2B–E) and/or OS (Figure 3B–E) according to changes in CD16+/CD56+ NK cells, CD16-/CD56- NK cells, NKp46-/CD56+ NK cells, and PD-1+ CD4+ T cells. A statistically significant difference in PFS was only shown between those with an increase in PD-1+ CD4+ T cells and those with a decrease in PD-1+ CD4+ T cells: 6.77 months (95% CI; 3.26 to 10.27 months) in patients with a decrease in PD-1+ CD4+ T cells vs. 2.57 months (95% CI: 0.74 to 4.40 months) in those with an increase in PD-1+ CD4+ T cells after treatment with ICIs (*p* = 0.010) (Figure 2E). There was significant difference in OS, especially between those with an increase of NK cells and those with a decrease of NK cells. OS was significantly longer in patients with an increase in CD16+/CD56+ NK cells than in those with a decrease in CD16+/CD56+ NK cells (16.77 months [95% CI: NR] vs. 7.63 months [95% CI: 4.20 to 11.06 months]) (*p* = 0.049) (Figure 3B). According to changes in CD16-/CD56- NK cells, the OS was significantly longer in patients with a decrease in CD16-/CD56- NK cells (16.77 months [95% CI: NR]) than in patients with an increase in CD16-/CD56- NK cells (5.63 months [95% CI: 2.58 to 8.69 months]) (*p* = 0.024) (Figure 3C). Patients with an increase in NKp46-/CD56+ NK cells showed significantly longer OS (16.77 months [95% CI: NR]), than those with a decrease in NKp46-/CD56+ NK cells (7.47 months [95% CI: 4.92 to 10.01 months]) (*p* = 0.013) (Table S2, Figure 3D). By changes of other immune cell fractions, there was no significant difference in OS (Table S2).

## 4. Discussion

ICIs have been widely used in cancer treatments. They have improved OS of patients with various cancer types, including metastatic melanoma and NSCLC [5,18]. Cancer cells can evade normal immune responses through multiple mechanisms, including upregulated immune checkpoints. Activation of checkpoint cascades such as those controlled by PD-1 or CTLA4 can result in inactivation of tumor-specific T cells and immune evasion [19,20]. Thus, treatment with anti-PD-1, anti-PD-L1, or anti-CTLA4 can reinvigorate T cells and make the adaptive immune system target cancer cells [21,22]. Nevertheless, not all patients can benefit from ICIs. There has been an unmet need for predicting the efficacy of ICIs. Many studies have been conducted to find a potential biomarker for treatment efficacy of ICIs. PD-L1 expression by IHC was approved by FDA as a diagnostic test for pembrolizumab in cancer treatment, including NSCLC, gastric or gastroesophageal junction adenocarcinoma, and urothelial carcinoma [23,24,25,26]. However, several studies have reported that not all patients with cancer expressing PD-L1 respond to immunotherapy [27,28]. TMB and dMMR/MSI-H have also been considered as predicting biomarkers for treatment efficacy of ICIs. However, there is still a debate about these candidates because the value could be different even within the tumor. In addition, the use of multiple assays and antibodies without a standardized framework for comparison or interpretation between assays makes extrapolation of findings from individual studies difficult and selection of clinical testing complicated [29].

In theory, tumor-infiltrating lymphocytes (TILs) are the main activator of antitumor immunity. TILs could be a promising biomarker if they could be objectively assessed throughout the whole tumor microenvironment. A previous study suggested that artificial intelligence-powered spatial analysis of TIL could be a complementary biomarker for ICIs in NSCLC [30]. However, there are practical difficulties in invasive procedures and analysis of lymphocytes in tissue. Thus, using peripheral blood for immune cell analysis has attracted attention. Peripheral blood absolute lymphocyte count has been used as a predictive biomarker [17]. To the best of our knowledge, studies investigating changes in immune cell fractions as a predictive biomarker for treatment efficacy of ICIs have not been reported yet. Thus, we conducted this pilot study to find out the potential of changes in immune cell fractions as a biomarker for predicting the efficacy of ICIs in cancer treatment.

During early stages of tumor development, cytotoxic immune cells such as NK and CD8+ T cells can recognize and eliminate more immunogenic cancer cells [31]. However, selected cancer cells can survive and progress to clinically detectable tumors that adopt different strategies of peripheral immune tolerance and recruitment of immunosuppressive immune cells [32]. Regulatory T cells (CD4+ T cells) also play a critical role in tumor progression by suppressing cytotoxic CD8+ T cell proliferation and favoring cancer cells escape from immunosurveillance [33]. Based on it, we thought that changes in fractions of NK cells, CD8+ T cells, and CD4+ T cells might be related to response to ICIs. However, results revealed that changes in fractions of CD4+ cells and CD8+ T cells were not related to the efficacy of ICIs. Patients with decreased CD8+ T cells tended to have higher response rates and longer survival. However, these results were statistically insignificant (Tables S1 and S2). It is thought that not only CD8+ T cells, but also other immune cells are related to the anti-tumoral effect. Even in panel 2 analysis, there was no T cell subtype that affected the response rate to ICIs (Table S1). In most patients, PD-1+ CD4+ T cells (*n* = 77/90, 85.6%) and PD-1+ CD8+ T cells (*n* = 73/90, 81.1%) were decreased. Among immune cell subtypes analyzed in this study, median change value for these cells were the highest: −5.49% (range, −30.60% to 24.77%) and −5.31% (range, −31.47% to 42.57%), respectively (Table 2). Response rate and survival results tended to be superior in patients with decreased PD-1+ CD4+ or PD-1+ CD8+ T cells. However, only PD-1+ CD4+ T cells showed a statistically significant the relationship in PFS. Patients with a decrease of PD-1+ CD4+ T cell fraction had a PFS of 6.77 months (95% CI: 3.26 to 10.27 months) and patients with an increase of PD-1+ CD4+ T cell fraction had a PFS of 2.57 months (95% CI: 0.74 to 4.40 months) (*p* = 0.010) (Table S2, Figure 2E).

NK cells are crucial components of the innate immune system owing to their early production of cytokines and chemokines and their ability to lyse target cells without prior sensitization. Human NK cells, comprising up to 15% of all circulating lymphocytes, can be divided mainly into two subsets based on their cell-surface density of CD56 (CD56- and CD56+), each with distinct phenotypic properties. The CD56- NK cell subset is more naturally cytotoxic. It expresses higher levels of Ig-like NK receptors and FCγ receptor III (CD16) than the CD56+ NK cell subset. By contrast, the CD56+ subset has the capacity to produce abundant cytokines following activation of monocytes. It has lower natural cytotoxicity and expresses lower levels of CD16 or is CD16- [34]. NKp46 is a type I transmembrane glycoprotein, expressed by all resting or activated NK cells, but not on T cells, B cells, granulocytes, monocytes, dendritic cells, or macrophages. Receptor triggering can lead to Ca^2+^ induction, driving not only perforin-mediated cytotoxicity but also secretion of inflammatory cytokines, mainly interferon-γ and tumor necrosis factor (TNF)-α [35,36]. CD56, CD16, and NKp46, NK cells all express a variegated pattern of inhibitory and activating receptor. The net sum of these signals adjusts the cytotoxic response of NK cells [37]. On this basis, changes in fraction of NK cells subgroups were also analyzed in this study.

Results of this study revealed that the increase of CD16+/CD56+ NK cell fraction and the decrease of CD16-/CD56- NK cell fraction were significantly related to longer OS (*p* = 0.049 and *p* = 0.024, respectively) (Table S2, Figure 3B,C). However, according to changes in CD16+/CD56+ NK cell fraction and CD16-/CD56- NK cell fraction, there were no significant differences in ORR (*p* = 0.205 and *p* = 0.072, respectively), CBR (*p* = 0.375 and *p* = 0.387, respectively), and PFS (*p* = 0.102 and *p* = 0.113, respectively) (Tables S1 and S2, Figure 2B,C). An increase in the fraction of NKp46-/CD56+ NK cells was related to a higher ORR (22.0% versus 5.0%, *p* = 0.033) (Table S1) and a longer OS (16.77 [95% CI: NR] months vs. 7.47 [95% CI: 4.92 to 10.01] months, *p* = 0.013) (Table S2, Figure 3D). PFS also tended to be longer in patients with an increase in NKp46-/CD56+ NK cells, although the difference was not statistically significant (6.97 [95% CI: 4.91 to 9.02] months vs. 3.30 [95% CI: 2.13 to 4.47] months, *p* = 0.050) (Table S2, Figure 2D). A previous study has analyzed results of 13 randomized trials with ICIs and shown a weak association between PFS/ORR and OS, although responders tend to exhibit longer survival [38]. Given that the ultimate goal of cancer treatment is to achieve survival benefit, it is thought that the OS could represent treatment outcomes. Based on this, it is thought that changes in NKp46-/CD56+ NK cell fraction might be a predicting biomarker for treatment efficacy of ICIs. However, the mechanisms involved in the changes in cell fraction and the anti-tumor effect are currently unknown, emphasizing further comprehensive research.

This study has several limitations. First, the type of cancer, treatment lines, and previously used drugs in each patient were various, which might have affected results of treatment. Second, response to treatment was assessed using RECIST, not immune-based therapeutics RECIST. Thus, pseudo-progression to ICIs might have been missed. Third, blood sampling was not carried out for patients with early death. Thus, the relationship between early death and immune cell fraction could not be analyzed. Fourth, only Asian patients were enrolled in this study, limiting the generalizability of study results. Nevertheless, this study is the first effort to analyze changes in immune cell fraction before and after immunotherapy. Results showed a possibility of using changes in immune cell fraction, especially NKp46-/CD56+ NK cells, as a predictor for treatment effect of ICIs. Further prospective clinical trials are needed to determine whether immune cell fraction changes could be a novel predictive biomarker for efficacy of ICIs in cancer treatment.

## 5. Conclusions

Changes in immune cell fraction before and after ICI administration could be a novel biomarker for predicting the treatment efficacy and survival benefit of ICIs. However, further study is needed to determine the outstanding immune cell type, the mechanism, and clinical correlations.

## Figures and Tables

**Table 1:** Baseline characteristics of patients.

Variables (*n* = 90)	No. of Patients (%)
Median age (years) (range)	65.0 (26 to 84)
Sex	
Male	55 (61.1%)
Female	35 (38.9%)
ECOG performance status	
0	10 (11.1%)
1	78 (86.7%)
2	2 (2.2%)
Type of cancer	
Lung cancer	45 (50.0%)
Others ^†^	45 (50.0%)
Previous lines of treatment	
0	13 (14.4%)
1	41 (45.6%)
2	22 (24.4%)
≥3	14 (15.6%)
Immune checkpoint inhibitor	
Pembrolizumab	36 (40.0%)
Nivolumab	40 (44.4%)
Atezolizumab	14 (15.6%)
Durvalumab	-
Combined chemotherapy	
Yes ^‡^	2 (2.2%)
No	88 (97.8%)

Others ^†^ include cholangiocarcinoma (*n* = 7), hepatocellular carcinoma (*n* = 6), gastroesophageal cancer (*n* = 6), head and neck cancer (*n* = 4), urothelial carcinoma (*n* = 4), colon cancer (*n* = 3), melanoma (*n* = 3), renal cell carcinoma (*n* = 3), ovarian cancer (*n* = 2), anal cancer (*n* = 1), Hodgkin lymphoma (*n* = 1), mesothelioma (*n* = 1), osteosarcoma (*n* = 1), pancreas cancer (*n* = 1), thymic carcinoma (*n* = 1), and uterine cancer (*n* = 1). ^‡^ Pemetrexed plus carboplatin was used with pembrolizumab in two patients with lung cancer.

**Table 2:** The change of the immune cell fraction.

Type of Immune Cells	Median Value of Change (%) (Range)
Panel 1	
CD4+ T cell	−1.95 (−36.20 to 31.60)
CD8+ T cell	1.55 (−27.40 to 32.50)
CD14+ monocyte	−0.51 (−34.90 to 43.60)
CD19+ B cell	−0.27 (−6.90 to 10.38)
Q1: CD16−/CD56+	−0.45 (−17.20 to 11.31)
Q2: CD16+/CD56+	4.05 (−37.10 to 48.58)
Q3: CD16+/CD56−	0.33 (−18.80 to 11.17)
Q4: CD16−/CD56−	−4.00 (−49.60 to 42.80)
Q5: NKp46−/CD56+	0.30 (−24.80 to 20.50)
Q6: Kp46+/CD56+	1.45 (−28.14 to 33.90)
Q7: NKp46+/CD56−	0.12 (−3.25 to 4.24)
Q8: NKp46−/CD56−	−4.60 (−51.60 to 43.10)
Panel 2	
PD-1+ CD4+	−5.49 (−30.60 to 24.77)
CTLA4+ CD4+	1.25 (−12.30 to 27.80)
CD39+ CD4+	0.29 (−6.50 to 12.71)
Ki-67+ CD4+	0.45 (−8.66 to 6.90)
GrB+ CD4+	0.96 (−26.82 to 24.30)
PD-1+ CD8+	−5.31 (−31.47 to 42.57)
CTLA4+ CD8+	0.71 (−13.10 to 32.20)
CD39+ CD8+	0.37 (−19.67 to 17.11)
Ki-67+ CD8+	0.53 (−14.80 to 20.60)

PD-L1, programmed cell death ligand-1; CTLA4, cytotoxic T-lymphocyte-associated protein 4; GrB, granzyme B.

**Table 3:** Response and survival results.

Variables (*n* = 90)	Value
Best response	
CR	-
PR	13 (14.4%)
SD	32 (35.6%)
PD	34 (37.8%)
Unknown	11 (12.2%)
ORR	13 (14.4%)
CBR	45 (50.0%)
Median treatment cycle (range)	5 (1 to 28)
Median treatment duration (months) (IQR)	2.40 (1.31 to 5.53)
Median PFS (months) (95% CI)	6.00 (3.11 to 8.89)
Median OS (months) (95% CI)	13.90 (5.62 to 22.19)

CR, complete response; PR, partial response; SD, stable disease; PD, progressive disease; ORR, objective response rate; CBR, clinical benefit rate; IQR, interquartile range; PFS, progression-free survival; CI, confidence interval; OS, overall survival.

## Data Availability

The data presented in this study are available on request from the corresponding author.

## Supplementary Materials

The following supporting information can be downloaded at https://www.mdpi.com/article/10.3390/cancers14143440/s1, Table S1: Immune cell analysis and response rate; Table S2: PFS and OS (months) (95% CI) according to the changes in the immune cell fraction.

## Appendix A

The procedure and protocol for performing immune cell analysis:

**Procedure**

**1. Preparation of Buffer and Reagents.**

1.1. Fixable Viability Stain 780.

1.1.1. Store the stock vial of FVS 780 desiccated and protected from light at −80 °C until use. Add 180 µL of DMSO into the vial of FVS 780 and vortex solution well. Inspect the solution and repeat vortex until the stock dye has fully dissolved. After reconstitution with DMSO, store the reconstituted dye in aliquots of 20 µL at −20 °C protected from light.

**2. Assay Controls.**

2.1. Compensation controls.

2.1.1. Add 100 µL of Staining Buffer (FBS) to each tube.

2.1.2. Add 1 full drop (approximately 60 µL) of the BD CompBead Anti-Mouse Ig, k (BD552843) beads to each tube and vortex.

2.1.3. Add antibody to the labeled tube according to Table A1 below:

**Table A1:** Compensation beads.

	Antibody Name	Catalog No.	Antibody Volume (µL)
**Panel 1**	CD4-BV421	562,424	0.3 (T4)
	CD3-BV510	564,713	0.6 (T3)
	CD8-BV650	563,821	0.3 (T4)
	CD14-FITC	347,493	0.2 (1:100)
	NKp46-PE	557,991	5 (T2)
	CD19-BB700	566,397	0.6 (T3)
	CD56-PE-Cy7	557,747	1.2 (T2)
	CD16-AF647	557,710	0.05 (1:100)
**Panel 2**	CD4-BV421	562,424	0.3 (T4)
	CD3-BV510	564,713	0.6 (T3)
	CD8-BV650	563,821	0.3 (T4)
	PD-1-FITC	557,860	2 (T1)
	CTLA4-PE	555,853	5 (T2)
	CD39-PerCP-Cy5.5	564,899	1.2 (T2)
	Ki-67-PE-Cy7	561,283	1.2 (T2)
	GranzymeB-AF647	560,212	0.1 (1:50)

2.1.4. Incubate 20 min at RT. Protect from exposure to direct light.

2.1.5. Add 1 mL Staining Buffer to each tube and pellet by centrifugation at 500× *g* for 5 min.

2.1.6. Discard supernatant from each tube by careful pipetting.

2.1.7. Resuspend bead pellet in each tube by adding 0.3 mL of Staining Buffer to each tube and transfer to each 12 × 75 mm tube. Vortex thoroughly.

2.2. FMO control.

2.2.1. Follow to the Step 4 Sample Staining.

**3. Cell Preparation.**

3.1. Thaw and count the frozen PBMC cells. Refer to LA-1-XXX, *Analyzing Count and Viability of PBMC Using Muse Cell Analyzer*. Record on the form LA-6-XXX.

**4. Sample Staining.**

4.1. Bring the buffers to RT before use.

4.2. Pipette 1 × 10^6^ PBMC per 2 mL microtube in 100 µL Staining Buffer (FBS).

4.3. Kill PBMC with DMSO in FVS780 compensation single control tube only.

4.3.1. Pipette 50 µL of DMSO into FVS780 compensation single control tube.

4.3.2. Incubate at RT for 20 min.

4.3.3. After the incubation period, add 100 µL of PBS to the tube.

4.3.4. Centrifuge the tube at 800× *g* for 5 min, at 4 °C.

4.3.5. Gently pipette off the supernatant and resuspend the pellet in 1 mL PBS.

4.4. Resuspend FMO control and sample tubes in 1 mL PBS.

4.5. Stain all tubes excluding Unstained PBMC compensation control tube with 1:1000 dilution of FVS 780 (add 1 µL of dye into 1 mL PBS).

4.6. Incubate for 10 min RT. Protect from exposure to direct light.

4.7. Centrifuge 450× *g* for 5 min, and remove solution.

4.8. Wash cells 1 time with 1 mL of Staining Buffer (FBS). Centrifuge the tubes at 450× *g* for 5 min.

4.9. Decent the supernatant and gently mix to disrupt the cell pellet.

4.10. Resuspend the cells in 50 µL BD Horizon Brilliant Stain Buffer and add 50 µL Stain Buffer (FBS).

4.11. Pipette appropriate amount of surface staining reagent to bottom of each 1.5 microtube according to Table A2 and Table A3 below:

**Table A2:** Amount of Surface staining reagents for panel 1.

Panel 1	Surface Stains								
	FVS 780 (565,388)	CD3 BV510 (564,713)	CD4 BV421 (562,424)	CD8 BV650 (563,821)	CD14 FITC (347,493)	NKp46 PE (557,991)	CD19 BB700 (566,396)	CD56 PE-Cy7 (557,747)	CD16 AF647 (557,710)
1. Unstained PBMC compensation control	NA	NA	NA	NA	NA	NA	NA	NA	NA
2. Killed PBMC FVS780 viability compensation control	1:1000	NA	NA	NA	NA	NA	NA	NA	NA
3. NKp46 FMO	1:1000	0.6 µL	0.3 µL	0.3 µL	10 µL *	NA	0.6 µL	1.2 µL	2.5 µL *
4. CD16 FMO	1:1000	0.6 µL	0.3 µL	0.3 µL	10 µL *	5 µL	0.6 µL	1.2 µL	NA *
5. CD56 FMO	1:1000	0.6 µL	0.3 µL	0.3 µL	10 µL *	5 µL	0.6 µL	NA	2.5 µL *
6. All Stains	1:1000	0.6 µL	0.3 µL	0.3 µL	10 µL *	5 µL	0.6 µL	1.2 µL	2.5 µL *

* 50× dilution done.

**Table A3:** Amount of Surface and intracellular staining reagents for panel 2.

Panel 2	Surface Stains						Intracellular Stains		
	FVS 780 (565,388)	CD3 BV510 (564,713)	CD4 BV421 (562,424)	CD8 BV650 (563,821)	PD-1 FITC (557,860)	CD39-PerCP-Cy5.5 (564,899)	CTLA4 PE (555,853)	Ki-67-PE-Cy7 (561,283)	GranzymeB-AF647 (560,212)
1. Unstained PBMC compensation control	NA	NA	NA	NA	NA	NA	NA	NA	NA
2. Killed PBMC FVS780 viability compensation control	1:1000	NA	NA	NA	NA	NA	NA	NA	NA
3. PD-1 FMO	1:1000	0.6 µL	0.3 µL	0.3 µL	NA	1.2 µL	5 µL	1.2 µL	5 µL *
4. CD39 FMO	1:1000	0.6 µL	0.3 µL	0.3 µL	2 µL	NA	5 µL	1.2 µL	5 µL *
5. CTLA4 FMO	1:1000	0.6 µL	0.3 µL	0.3 µL	2 µL	1.2 µL	NA	1.2 µL	5 µL *
6. Ki-67 FMO	1:1000	0.6 µL	0.3 µL	0.3 µL	2 µL	1.2 µL	5 µL	NA	5 µL *
7. Granzyme B FMO	1:1000	0.6 µL	0.3 µL	0.3 µL	2 µL	1.2 µL	5 µL	1.2 µL	NA *
8. All Stains	1:1000	0.6 µL	0.3 µL	0.3 µL	2 µL	1.2 µL	5 µL	1.2 µL	5 µL *

* 50× dilution done.

4.12. Mix samples by a pulse vortex or gently flicking the tube.

4.13. Incubate for 30 min at 4 °C, protected from light.

4.14. Add 1 mL of Staining Buffer (FBS) to wash. Centrifuge 450× *g* for 5 min, and remove solution. Repeat wash once more and then remove wash buffer.

4.15. For Panel 1 tubes, proceed to Step 4.23 and store tubes protected from light at 4 °C until analysis

4.16. For Panel 2 tubes excluding FVS780 compensation control, to fix and permeabilize the cells, gently re-suspend pellet in residual volume of wash buffer and then add 250 µL for fixation/permeabilization solution. Vortex.

4.17. Incubate 20 min at 4 °C, protected from light.

4.18. To prepare working solutions of the BD Perm/Wash^TM^ Buffer (10X concentrate), dilute 1:10 in distilled H_2_O prior to use.

4.19. To wash cells, add 1 mL of 1X BD Perm/Wash^TM^ Buffer and centrifuge 500× *g* for 5 min. Remove solution. Repeat wash once more and then remove wash buffer.

4.20. Gently re-suspend pellet in 50 µL of 1X BD Perm/Wash^TM^ Buffer and add antibodies for intracellular stains in panel 2 at appropriate concentration according to Table A3 to re-suspend the pellet. Gently shake or vortex.

4.21. Incubate for 30 min in the dark at 4 °C.

4.22. Repeat wash as in Step 4.19.

4.23. Re-suspend in stain buffer and analyze immediately.

***Optional:*** Add 300 µL of 1% formaldehyde in 1 × PBS and store at 4 °C. Analyze cells within 24 h.
