# Supplementary material for: Changes of Immune Cell Fractions in Patients Treated with Immune Checkpoint Inhibitors
Source: Cancers (Basel). 2022 Jul 15;14(14):3440. doi: 10.3390/cancers14143440 (PMC9315861; doi:10.3390/cancers14143440)
Supplement: Supplementary file 1 [file cancers-14-03440-s001.zip › cancers-1801233-supplementary.pdf]

Table S1. Immune cell analysis and response rate

	ORR	*p*-value	CBR	*p*-value
Panel 1				
CD14+ monocyte				
Decrease (n=46)	15.2%	1.000	45.7%	0.527
Increase (n=44)	13.6%		54.5%	
CD19+ B cell				
Decrease (n=53)	13.2%	0.765	49.1%	1.000
Increase (n=37)	16.2%		51.4%	
CD4+ T cell				
Decrease (n=56)	14.3%	1.000	48.2%	0.828
Increase (n=34)	14.7%		52.9%	
CD8+ T cell				
Decrease (n=33)	18.2%	0.537	57.6%	0.382
Increase (n=57)	12.3%		45.6%	
Q1: CD16-/CD56+				
Decrease (n=52)	13.5%	0.770	50.0%	1.000
Increase (n=38)	15.8%		50.0%	
Q2: CD16+/CD56+				
Decrease (n=31)	6.5%	0.205	41.9%	0.375
Increase (n=59)	18.6%		54.2%	
Q3: CD16+/CD56-				
Decrease (n=41)	9.8%	0.368	46.3%	0.672
Increase (n=49)	18.4%		53.1%	
Q4: CD16-/CD56-				
Decrease (n=55)	20.0%	0.072	54.5%	0.387
Increase (n=35)	5.7%		42.9%	
Q5: NKp46-/CD56+				
Decrease (n=40)	5.0%	0.033	40.0%	0.137
Increase (n=50)	22.0%		58.0%	
Q6: NKp46+/CD56+				
Decrease (n=33)	12.1%	0.761	48.5%	1.000
Increase (n=57)	15.8%		50.9%	
Q7: NKp46+/CD56-				
Decrease (n=35)	14.3%	1.000	48.6%	1.000
Increase (n=55)	14.5%		50.9%	
Q8: NKp46-/CD56-				
Decrease (n=57)	19.3%	0.121	54.4%	0.382

Increase (n=33)	6.1%		42.4%	
Panel 2				
PD-1+ CD4+				
Decrease (n=77)	16.9%	0.201	54.5%	0.069
Increase (n=13)	0.0%		23.1%	
CTLA4+ CD4+				
Decrease (n=29)	13.8%	1.000	44.8%	0.652
Increase (n=61)	14.8%		52.5%	
CD39+ CD4+				
Decrease (n=35)	14.3%	1.000	48.6%	1.000
Increase (n=55)	14.5%		50.9%	
Ki-67+ CD4+				
Decrease (n=40)	17.5%	0.552	45.0%	0.525
Increase (n=50)	12.0%		54.0%	
GrB+ CD4+				
Decrease (n=28)	10.7%	0.747	53.6%	0.820
Increase (n=62)	16.1%		48.4%	
PD-1+ CD8+				
Decrease (n=73)	16.4%	0.448	52.1%	0.591
Increase (n=17)	5.9%		41.2%	
CTLA4+ CD8+				
Decrease (n=39)	12.8%	0.770	41.0%	0.202
Increase (n=51)	15.7%		56.9%	
CD39+ CD8+				
Decrease (n=29)	20.7%	0.336	55.2%	0.652
Increase (n=61)	11.5%		47.5%	
Ki-67+ CD8+				
Decrease (n=41)	17.1%	0.559	41.5%	0.204
Increase (n=49)	12.2%		57.1%	
GrB+ CD8+				
Decrease (n=26)	11.5%	0.749	53.8%	0.816
Increase (n=64)	15.6%		48.4%	

PD-1, programmed cell death protein 1; CTLA4, cytotoxic T-lymphocyte-associated protein 4; GrB, granzyme B.

Table S2. PFS and OS (months) (95% CI) according to the changes in the immune cell fraction

	Decrease	Increase	*p*-value
Progression-free survival			
CD14+ monocyte	6.00 (1.74-10.27)	4.93 (0.54-9.33)	0.558
CD19+ B cell	6.00 (2.38-9.62)	3.53 (0.00-7.28)	0.889
CD4+ T cell	6.00 (1.35-10.65)	6.57 (3.62-8.92)	0.722
CD8+ T cell	6.27 (1.82-10.71)	4.93 (0.55-9.32)	0.781
Q1: CD16- CD56+	4.67 (0.89-8.45)	6.33 (2.21-10.45)	0.562
Q2: CD16+ CD56+	3.30 (1.99-4.62)	6.97 (5.03-8.91)	0.102
Q3: CD16+ CD56-	4.67 (2.62-9.72)	6.27 (2.16-10.38)	0.902
Q4: CD16- CD56-	6.97 (5.08-8.85)	3.30 (1.94-4.66)	0.113
Q5: NKp46- CD56+	3.30 (2.13-4.47)	6.97 (4.91-9.02)	0.050
Q6: NKp46+ CD56+	3.53 (1.03-6.04)	6.33 (3.71-8.96)	0.765
Q7: NKp46+ CD56-	3.30 (2.63-3.97)	6.33 (3.77-8.90)	0.410
Q8: NKp46- CD56-	6.77 (5.28-8.26)	3.47 (2.37-4.57)	0.237
PD-1+ CD4+	6.77 (3.26-10.27)	2.57 (0.74-4.40)	0.010
CTLA4+ CD4+	3.53 (1.77-5.30)	6.97 (1.91-12.02)	0.606
CD39+ CD4+	3.53 (1.35-5.72)	6.77 (2.02-11.52)	0.261
Ki-67+ CD4+	6.00 (3.29-8.71)	3.53 (0.00-7.15)	0.957
GrB+ CD4+	6.97 (2.17-11.76)	3.50 (0.43-6.57)	0.263
PD-1+ CD8+	6.33 (2.76-9.91)	6.47 (0.75-6.19)	0.304
CTLA4+ CD8+	3.53 (0.45-6.92)	6.77 (3.21-10.33)	0.551
CD39+ CD8+	6.77 (3.51-10.03)	3.53 (0.74-6.33)	0.212
Ki-67+ CD8+	6.00 (2.45-9.55)	6.33 (2.17-10.50)	0.875
GrB+ CD8+	6.27 (1.05-11.48)	6.00 (2.21-9.79)	0.658
Overall survival			
CD14+ monocyte	16.77 (3.98-29.55)	13.90 (NR)	0.760
CD19+ B cell	13.90 (4.20-23.60)	NR	0.420
CD4+ T cell	7.63 (0.89-14.37)	NR	0.101
CD8+ T cell	NR	13.70 (3.83-23.57)	0.565
Q1: CD16- CD56+	13.90 (7.91-19.90)	13.70 (NR)	0.748
Q2: CD16+ CD56+	7.63 (4.20-11.06)	16.77 (NR)	0.049
Q3: CD16+ CD56-	7.47 (1.07-13.87)	16.77 (10.66-22.88)	0.159
Q4: CD16- CD56-	16.77 (NR)	5.63 (2.58-8.69)	0.024
Q5: NKp46- CD56+	7.47 (4.92-10.01)	16.77 (NR)	0.013
Q6: NKp46+ CD56+	7.63 (4.05-11.22)	16.77 (NR)	0.053
Q7: NKp46+ CD56-	13.90 (2.21-25.59)	NR	0.150

Q8: NKp46- CD56-	16.77 (NR)	7.63 (0.93-14.33)	0.082
PD-1+ CD4+	13.90 (10.42-17.38)	7.63 (0.00-16.62)	0.367
CTLA4+ CD4+	NR	13.70 (4.90-22.50)	0.243
CD39+ CD4+	13.70 (0.00-27.84)	16.77 (5.17-28.36)	0.955
Ki-67+ CD4+	NR	13.90 (3.73-24.07)	0.469
GrB+ CD4+	NR	13.70 (3.05-24.35)	0.295
PD-1+ CD8+	16.77 (12.34-21.19)	5.60 (0.00-11.38)	0.095
CTLA4+ CD8+	NR	13.70 (6.96-20.44)	0.408
CD39+ CD8+	NR	13.70 (3.76-23.64)	0.240
Ki-67+ CD8+	NR	13.70 (5.84-21.67)	0.122
GrB+ CD8+	13.90 (NR)	13.70 (3.82-23.58)	0.973

PFS, progression-free survival; OS, overall survival; CI, confidential index; PD-1, programmed cell death protein 1; CTLA4, cytotoxic T-lymphocyte-associated protein 4; GrB, granzyme B; NR, not reached.
